# Longitudinal Changes on Clinical Features in 28 Children With COVID-19 in Shenzhen, China

**DOI:** 10.3389/fmed.2020.579406

**Published:** 2020-11-04

**Authors:** Xuejiao Liao, Jiaye Liu, Ziyi He, Ming Hu, Tongyang Xiao, Lanlan Wei, Qiue Cai, Haiyan Wang, Qing He, Lei Liu, Zheng Zhang

**Affiliations:** ^1^The Third People's Hospital of Shenzhen, The Second Affiliated Hospital, School of Medicine, Southern University of Science and Technology, Shenzhen, China; ^2^Institute of Hepatology, Shenzhen Third People's Hospital, Shenzhen, China

**Keywords:** coronavirus disease 2019 (COVID-19), severe acute respiratory syndrome coronavirus 2 (SARS-CoV-2), pediatric, antibody, clinical features

## Abstract

**Objective:** To investigate the clinical characteristics of children with coronavirus disease 2019 (COVID-19) and identify the occurrence of viral shedding of severe acute respiratory syndrome coronavirus-2 (SARS-CoV-2) during follow-up.

**Methods:** We retrospectively retrieved data from pediatric patients with COVID-19 from the Shenzhen Third People's Hospital in China. The dynamics of SARS-CoV-2 and antibodies against SARS-CoV-2 were analyzed during hospitalization and after discharge.

**Results:** From January 23 to March 15, 2020, a total of 28 pediatric patients were diagnosed with COVID-19 and were followed for at least 1 month. The median age was 7 years (IQR 3.5–10) and none of the children progressed to severe COVID-19 during hospitalization. Ten patients tested positive for SARS-CoV-2 1 month after discharge while four patients tested positive during the 2nd month after discharge. Only three of 12 children showed detectable immunoglobulin-M (IgM) on day 5, 18, and 21 after illness onset, respectively.

**Conclusions:** COVID-19 disease was relatively mild among children while a number did test positive after discharge from the hospital. Public health initiatives should thus adapt control measures targeted toward children.

## Introduction

Coronavirus disease 2019 (COVID-19), a newly emerged respiratory disease caused by severe acute respiratory syndrome coronavirus 2 (SARS-CoV-2), has become a global pandemic. According to the “Coronavirus Disease 2019 (COVID-19) Situation Report” from the World Health Organization (WHO), nearly 20 million confirmed cases were reported globally as of August 10, 2020 (1). The current evidence confirms that people of all ages are susceptible to COVID-19, with the latest available information indicating that from January 16, 2020, to February 8, 2020, ~2, 135 pediatric infections occurred in China (2). With the growing COVID-19 pandemic, more children are at risk of infection and subsequent negative outcomes. Current studies however, have primarily focused on the epidemiological and clinical characteristics and outcomes of infected adults (3, 4). A general understanding of how COVID-19 presents in adolescents and the factors that may lead to more advanced disease in children needs to be understood.

In addition, the presence of antibodies in individuals with COVID-19 lacks conclusive evidence of a protective effect. A previous study determined that 93.1, 82.7, and 64.7% of adults infected with SARS-CoV-2 achieved sero-conversion of total antibody (Ab), immunoglobulin-G (IgG), immunoglobulin-A (IgA), and immunoglobulin-M (IgM) (5). Although the presence of these antibodies to SARS-CoV-2 as being protective or pathogenic has yet to be determined, the production of antibodies does provide support for the routine application of serological testing for the diagnosis and management of COVID-19 patients. To the best of our knowledge, first of all, no other investigations have thus far reported on the dynamic changes of antibodies among pediatric patients. Second, only one study from China found three pediatric cases that tested SARS-CoV-2 positive in the stool samples of patients within 10 days after discharge, even though these patients tested negative for nucleic acid when using throat swab specimens. This implies that children who have recovered might still be possible carriers for the virus (6). Finally, no clinical data have been reported on children who have been followed up for more than 2 months. Thus, it is necessary to investigate the occurrence of antibodies and reoccurrence of SARS-CoV-2 among children who have recovered from COVID-19. Further robust analysis of pediatric patients from multi-centers is still needed in order to better understand how the disease presents in children, what associated outcomes may be linked to COVID-19, and in general, to help support the development of guidelines for the successful treatment and care of children infected with COVID-19 through cooperative researches. Hence, we aimed to investigate the clinical characteristics of children with COVID-19 and identify the occurrence of viral shedding of SARS-CoV-2 during follow-up.

## Methods

### Study Design and Participants

We conducted a retrospective review of medical records from 28 pediatric patients with confirmed COVID-19 pneumonia admitted to the Shenzhen Third People's Hospital in China from January 23, 2020 to March 15, 2020. All children were diagnosed according to the WHO interim guidance (7). Records were excluded if the patient was older than 15 years old or without baseline clinical characteristics. This study was reviewed and approved by the Medical Ethical Committee of the Shenzhen Third People's Hospital (approval number 2020-201). Written informed consent was obtained from the guardians of the children enrolled in this study.

### Confirmation of COVID-19 Infection

The presence of SARS-CoV-2 was detected by real-time reverse transcription polymerase chain reaction (RT-PCR) (8). Two pairs of primers targeting the open reading frame 1ab (ORF1ab) and the nucleocapsid protein (N) were amplified and examined. Each sample was run in triplicate with positive and negative controls set as suggested. Samples identified as positive for SARS-CoV-2 by the local laboratory were confirmed by the key laboratory of the Shenzhen Centers for Disease Control (CDC). These diagnostic criteria were based on the recommendations by the Chinese Center for Disease Control and Prevention (China CDC). All patients were classified as mild, ordinary, or severe cases based on results from chest radiography, clinical examinations, and symptoms (9).

### Data Collection and Follow-Up

Two physicians reviewed epidemiological, clinical, laboratory, and radiological characteristics and treatment outcomes using standard data collection forms from electronic medical records. Baseline was defined as the time of first hospital admission due to COVID-19 and all pediatric patients underwent routine SARS-CoV-2 nucleic acid testing every 3 days during hospitalization. Patients were discharged from the hospital after clearance of SARS-CoV-2. Viral clearance was defined by the presence of two consecutive negative results in qRT-PCR detection for SARS-CoV-2 RNA at an interval of 24 h, and the day of the first one of these two tests was considered as the clearance day. Within the 1st month after discharge from the hospital, all patients received at least four additional tests for the presence of SARS-Cov-2 RNA at day 3–7, 13–14, 20–22, and 28–30 after discharge from the hospital. After discharged from the hospital for patients who achieved clearance of SARS-CoV-2, they would be re-admitted to the hospital if they were tested positive for SARS-CoV-2 RNA during follow-up. Patients were categorized as the “early virus clearance group” if they had four consecutive negative results of qPCR detection for SARS-CoV-2 RNA within 1 month after they achieved two consecutive negative results of qPCR detection for SARS-CoV-2 RNA with an interval of 24 h. While patients who had any positive result of qPCR detection for SARS-CoV-2 RNA within the 1st month after two consecutive negative results of qPCR detection for SARS-CoV-2 RNA with an interval of 24 h were categorized as “delayed virus clearance group.”

### Antibody Testing

The SARS-CoV-2 specific Ab, IgG, IgA, and IgM in plasma was tested using a Chemiluminescence Microparticle Immuno Assay (CMIA). Briefly, antigens containing the receptor-binding domain (RBD) were used as the immobilized and horseradish peroxidase (HRP)-conjugated antigen to detect total antibodies by double-antigen sandwich enzyme-linked immunosorbent assay (Ab-ELISA). IgM was tested by the IgM μ-chain capture method (IgM-ELISA), using the same RBD antigen as the Ab-ELISA. IgA and IgG were tested by indirect ELISA using RBD antigen. The testing kits were provided by Beijing Wantai Biological Pharmacy Enterprise Co., Ltd. Fluorescence intensity was used to measure antibody concentration. The relative fluorescence of sample to control (COI) was used to estimate the result. When COI was more than one, the result was judged to be positive.

### Statistical Analysis

Categorical variables were described as proportions and continuous variables were described using median and interquartile range (IQR) values. The Mann-Whitney test was used to compare the median for continuous variables. The Fisher exact test was used when the data were limited. All significance tests performed were two-sided. *P*-values <0.05 were deemed statistically significant. We also analyzed the longitudinal changes in clinical characteristics of 10 pediatric patients at admission to the hospital, at discharge from the hospital, and at re-admission to hospital due to reoccurrence of SARS-CoV-2. All analyses were carried out using SAS software version 9.4 (SAS Institute).

## Results

### Demographic, Epidemiological, and Clinical Characteristics

From January 23 to March 15, 2020, a total of 28 hospitalized, pediatric patients were diagnosed with COVID-19 and were followed-up for at least 1 month. As seen in Table 1, the median age was 7 years (IQR 3.5–10), and 18 (64.3%) were female. Twenty-seven (96.4%) patients had a close contact with confirmed COVID-19 patients. The median interval between illness onset and admission to the hospital was 1 day (IQR 0–1). Of the participants, 18 (64.3%) presented symptoms at admission to the hospital. The most common symptoms at onset of illness were fever (39.3%), cough (35.7%), and nasal discharge (10.7%). Six (21.4%) patients remained asymptomatic until discharge, while four patients had pulmonary computed tomography (CT) abnormalities, mostly appearing as frosted glass and nodules. Twenty-five patients received interferon-alpha treatment, 19 received antiviral therapy with Lopina-velitonavir, and 14 received treatment with probiotics. None of the children progressed to severe COVID-19. Of the 28 patients with COVID-19, more were older than 7 years (*n* = 10, *p* < 0.001) and the majority had close contact with a confirmed COVID-19 patient (*n* = 15, *p* = 0.035).

**Table 1:** Clinical features of 28 pediatric patients with COVID-19 at the Third People's Hospital of Shenzhen.

	**Total** **(*n*,%)**	**<7 years** **(*n*,%)**	**≥7 years** **(*n*,%)**	***P*-value**
Total number of case	28	13	15	
Age, median (IQR), years	7 (3.5, 10)	2 (2, 5)	10 (7,12)	0.000
Sex				
Male	10 (35.7)	5 (38.5)	5 (33.3)	1.000^*^
Female	18 (64.3)	8 (61.5)	10 (66.7)	
Close contact with confirmed COVID-19 patients	27 (96.4)	12 (92.3)	15 (100.0)	0.206^*^
Number of Days between illness onset and admission to hospital	1 (0, 1)	1 (0, 1)	1 (0, 1)	0.486
Symptoms				
Fever	11 (39.3)	6 (46.2)	5 (33.3)	0.488
Cough	10 (35.7)	6 (46.2)	4 (26.7)	0.433^*^
Nasal discharge	3 (10.7)	2 (15.4)	1 (6.7)	0.583^*^
Severity of illness				
Ordinary	24 (85.7)	13 (100%)	11 (83.3)	0.102^*^
Mild	4 (14.3)	0 (0.0)	4 (26.7)	
Asymptomatic^#^	6 (21.4)	3 (23.1)	3 (20.0)	1.000^*^
Radiological features of CT in Asymptomatic patients				
Bilateral pneumonia	3 (50.0)	1 (33.3)	2 (66.7)	1.000^*^
Unilateral pneumonia	1 (16.7)	1 (33.3)	0 (0.0)	0.464^*^
Frosted glass	2 (33.3)	1 (33.3)	1 (33.3)	1.000^*^
Nodules	1 (16.7)	0 (0.0)	1 (33.3)	1.000^*^
Normal	2 (33.3)	1 (33.3)	1 (33.3)	1.000^*^
Treatment				
Lopina-velitonavir	19 (67.9)	8 (61.5)	11 (73.3)	1.000^*^
Interferon atmotherapy	25 (89.3)	11 (84.6)	14 (93.3)	1.000^*^
Probiotics	14 (50.0)	6 (46.2)	8 (53.3)	1.000^*^

* *P-values were obtained by Fisher exact test. IQR, interquartile range; CT, computed tomography*.

# *Asymptomatic was defined as no clinical discomfort in the course of the disease*.

### SARS-CoV-2 Test Results Among 10 Pediatric Patients Whom Tested Positive During Follow-Up

**Figure 1:**
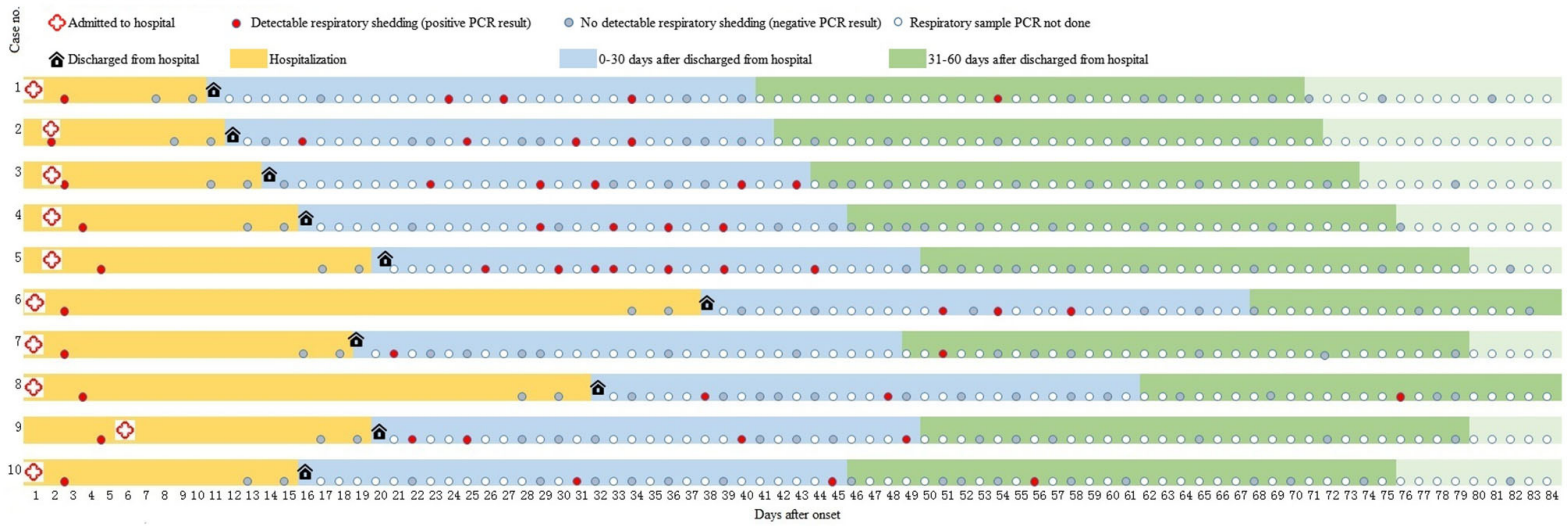
A summary of illness onset, hospitalization, and test results of 10 participants who tested positive for SARS-CoV-2 during follow-up. PCR, polymerase chain reaction.

A total of 10 patients tested SARS-CoV-2 positive at least one time, and 9 (90%) had more than two SARS-Cov-2 positive tests within 30 days after being discharged from the hospital, as seen in Figure 1. Eight patients completed 60 days of follow-up after being discharged from the hospital. During 31–60 days after discharge, four patients tested negative, while the other four patients tested positive on day 51, day 54, day 56, and day 76 after illness onset, respectively (Figure 1).

### Clinical Characteristics of the 10 Patients That Tested Positive During Follow-Up

The most common symptoms at admission to the hospital were fever (40%), cough (30%), and nasal discharge (20%). None of patients had fever and nasal discharge at discharge from the hospital, while three patients had cough during the whole course of their diseases.

At the first time admission to hospital, various proportions (10–60%) of patients had abnormal laboratory tests (Table 2). Three patients maintained lymphocytosis throughout the course of their hospitalization, and three patients had increasing alkaline phosphatase at re-admission to hospital compared to the first time of admission to hospital. Beside lymphocyte and alkaline phosphatase, other laboratory testing remained normal.

**Table 2:** Clinical characteristics of the 10 that tested positive for SARS-CoV-2 during follow up.

	**Admission to the hospital**	**Discharge from the hospital**	**Re-admission to the hospital**
Symptoms			
Fever	4 (40.0)	0 (0.0)	0 (0.0)
Cough	3 (30.0)	3 (30.0)	3 (30.0)
Nasal discharge	2 (20.0)	0(0.0)	0 (0.0)
Asymptomatic	2 (0.0)	7 (70)	7 (70.0)
Laboratory examination^*^			
Abnormal white blood cell count	2 (20.0)	0 (0.0)	0 (0.0)
Neutropenia	2 (20.0)	0 (0.0)	0 (0.0)
Lymphocytosis	3 (30.0)	3 (30.0)	3 (30.0)
Increasing aspartate aminotransferase	1 (10.0)	0 (0.0)	0 (0.0)
Increasing alkaline phosphatase	6 (60.0)	/	9 (90.0)
Increasing lactate dehydrogenase	1 (10.0)	0 (0.0)	0 (0.0)
Procalcitonin	3 (30.0)	0 (0.0)	0 (0.0)
Increasing serum interleukin-6 (IL-6) level	1 (10.0)	0 (0.0)	0 (0.0)
Increasing serum c-reactive protein (CRP) level	2 (20.0)	0 (0.0)	0 (0.0)
Chest radiographic imaging			
Normal	2 (20.0)	2 (20.0)	2 (20.0)
Bilateral pneumonia	4 (40.0)	4 (40.0)	4 (40.0)
Unilateral pneumonia	4 (40.0)	4 (40.0)	4 (40.0)
Frosted glass	2 (20.0)	3 (30.0)	0 (0.0)
Nodules	4 (40.0)	5 (50.0)	3 (30.0)
Treatment			
Antiviral therapy	7 (70.0)	0 (0.0)	0 (0.0)
Interferon atmotherapy	10 (100.0)	0 (0.0)	0 (0.0)
Traditional Chinese medicine	2 (20.0)	0 (0.0)	6 (60.0)
Probiotics	7 (70.0)	0 (0.0)	7 (70.0)

* *Normal range of laboratory examinations: white blood cell count: 5–12 × 10^9^ /L. neutrophil count is 1.8–6.3 × 10^9^ /L. lymphocyte count: 1.1–3.2 × 10^9^ /L. aspartate aminotransferase: 21–72 U/L. alkaline phosphatase: 38–126 U/L. lactate dehydrogenase: 313–618 U/L. Procalcitonin: <0.01 ng/ml. IL-6: <7 pg/ml. CRP: <8 mg/L*.

At the time of admission, a total of eight cases had ground glass and nodular changes on their CT scans. At the time of discharge and at the re-admission to hospital, the lesions were not completely absorbed, with a few nodules and inflammatory lesions remaining.

All 10 children received interferon nebulization therapy during their hospitalization, and seven of them received antiviral therapy. However, antiviral therapy was not continued after discharge. After re-admission to the hospital, 60% of patients received traditional Chinese medicine and 70% of patients received probiotics.

### Anti-SARS-CoV-2 IgG and IgM Antibodies in Pediatric Patients With COVID-19

**Figure 2:**
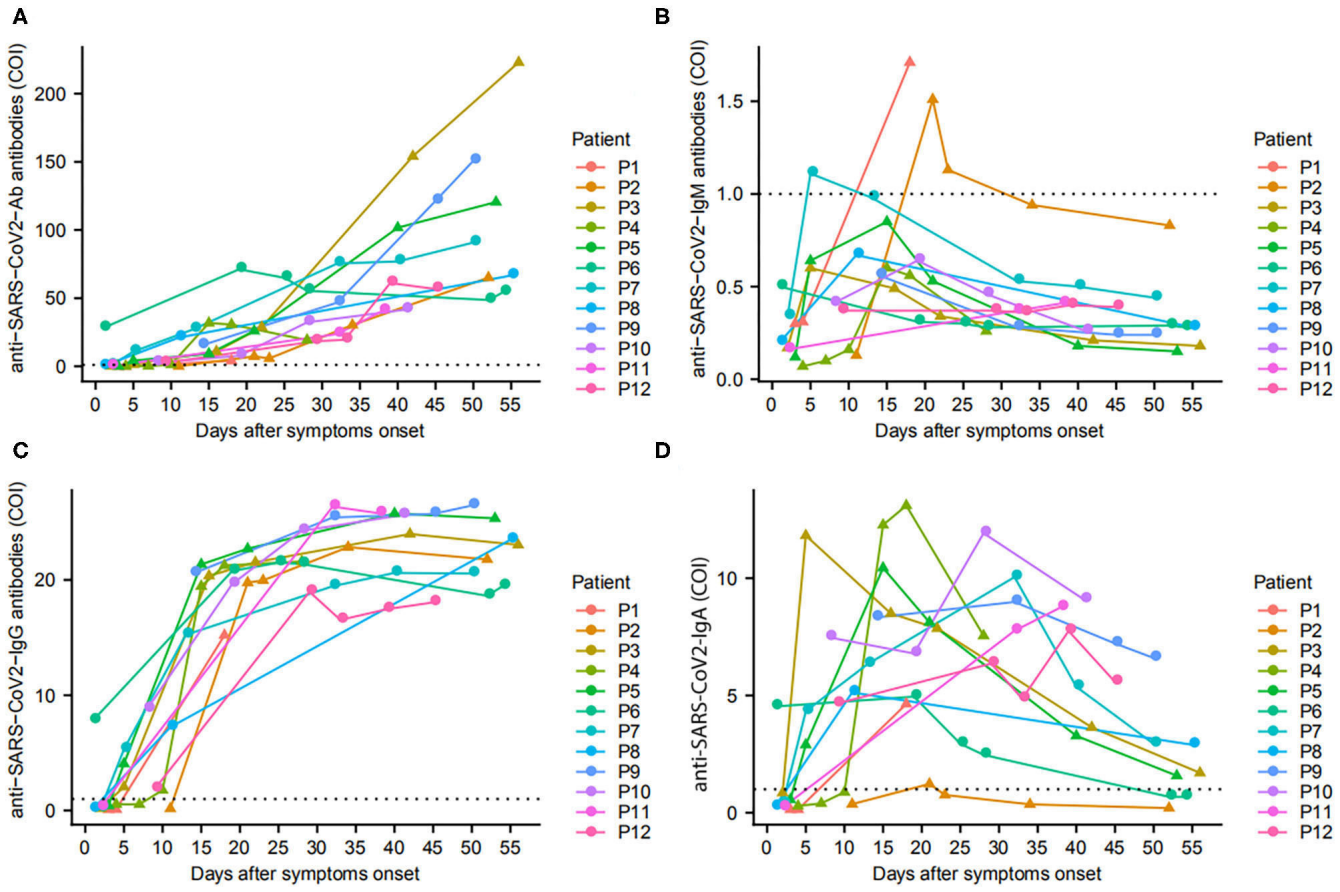
Antibodies in pediatric COVID-19 patients since onset of disease. The changes of total Ab **(A)**, IgM **(B)**, IgG **(C)**, and IgA **(D)** in 12 patients are presented. ▴ represents patients with early virus clearance, while • represents delayed virus clearance. …means cutoff values for antibody tests.

Of 28 pediatric patients, 12 patients tested antibodies against SARS-CoV-2 with a total of 57 plasma samples collected throughout the course of their diseases. The cumulative sero-conversion rate for Ab, IgM, IgA, and IgG was 100.0% (12/12), 25.0% (3/12), 100.0% (12/12), and 100.0% (12/12) during the follow-up period. Within 7 days of illness onset, the sero-conversion rate for Ab, IgA, and IgG was 66.7% (4/6), 66.7% (4/6), and 66.7% (4/6), while all these corresponding rates increased to 100% (9/9) on day 8–14 after onset, and maintained positive until day 30. In addition, we found only three children sero-converted IgM at day 5, day 18, and day 21 after disease onset, respectively (Figure 2).

## Discussion

There has been a substantial gap in understanding the course and pathogenic features of SARS-CoV-2 infection, especially for pediatric patients. In this study, we analyzed the clinical characteristics and antibody responses to SARS-CoV-2 infection to understand its impact on pediatric patients. This study observed that COVID-19 was relatively mild in pediatric patients, mainly representing non-occurrence of severity of disease and transient abnormal laboratory testing. Moreover, this study revealed that the reoccurrence of SARS-CoV-2 was common in pediatric patients, even during the 2nd month of follow-up after being discharged from the hospital. In addition, this study found a high sero-conversion rate of antibodies against SARS-CoV-2.

Investigation of SARS-CoV-2 shedding will be the key for determining the risk of transmission and formulating the criteria for being released from quarantine. In this study, the criteria for being discharged from the hospital were the presence of two consecutive negative results using qPCR detection for SARS-CoV-2 RNA within 24 h. However, 10 patients had reoccurrence of SARS-CoV-2 RNA during the 1st month of follow-up, and four patients tested positive for SARS-CoV-2 at the 2nd month of after being discharged from the hospital. Various reoccurrence rates of SARS-CoV-2 have also been reported in pediatric and adult patients in other studies (6, 10, 11). However, the study showed that the positive rate of SARS-CoV-2 after discharge in adult group was significantly lower than that in children in this study (12). Although it has not been confirmed whether patients with reoccurrence of SARS-CoV-2 are contagious, the high frequency of reoccurrence of SARS-CoV-2 implies that the virus may be transmitting, even among patients being released from quarantine. However, based on the findings in this study which showed fewer individuals testing positive for the disease 2 months after discharge, viral clearance could be completed within longer period than the suggested quarantine time. Further studies are needed to explore the interaction of host and SARS-CoV-2.

We also found pediatric patients had relatively milder clinical symptoms and laboratory abnormalities at admission to the hospital, which was consistent with several other studies (13, 14). Particularly, none of pediatric patients progressed to severe COVID-19, which was much lower than that in adult patients (15). The large proportion of children with asymptomatic or mild symptoms may lead to larger public health uncertainty, as it could be difficult to identify pediatric patients, resulting in an increased risk of intra-family transmission and general on-going transmission in China. Thus, though severe disease manifestation is not as expected in this specific patient population, public health messaging should still be adamant about the proper prevention and management strategies in children. Age appropriate resources and support for parents should be considered when updating national care and treatment guidelines.

Compared to PCR, serological testing is advantageous with faster turn-around time, high-throughput, and less workload. Based on the accumulated understanding of host antibody responses during infection, the presence of serum IgM and IgG antibody against SARS-CoV-2 has been added as one confirmation criteria of SARS-CoV-2 infection (16). Long et al. (17) reported that within 19 days after symptom onset, all 285 patients who were tested were positive for IgG against SARS-CoV-2. This study found that at 8–14 days after illness onset, 100% of pediatric patients tested positive for Ab, IgA, and IgG, suggesting that pediatric patients may achieve sero-conversion earlier than adult patients. A previous study showed a strong positive correlation between clinical severity and antibody titer 2 weeks after illness onset (5), but did not assess this causal relation as all patients presented with mild disease. Whatsoever, this study provides important evidences that total antibody and IgG antibody could be used to understand the epidemiology of SARS-CoV-2 infection, to assist in diagnosing and managing COVID-19 pediatric patients.

In addition, we found that the positive rate of IgM antibody in children was only 25% during follow-up, which was significantly lower than the result of adults in our previous study (5). The reason of the disparity between children and adults remains unclear. The positive rate of IgM antibody might varies in different virus. The low positive rate of IgM antibody also exists in children infected with influenza A, but it can reach 41–51% after influenza B infection (18). Therefore, it is necessary to collect large samples and regular data with dense detection time-points to explore the positive results of IgM antibody.

There were some limitations in this study. First, the sample size was small and the study was a case series. Though these findings may be valuable early data and add to the existing literature, the results may not be generalizable to the entire infected population. Second, serum samples were collected from 12 individuals to assess the dynamics of antibodies against SARS-CoV-2. The results may be biased to sample selection. Third, the influence of reoccurring SARS-CoV-2 virus on disease progression and transmission has not yet been confirmed. Further longitudinal studies assessing these factors in pediatric patients could help improve diagnostic and treatment criteria in this unique population.

## Conclusions

Among the 28 pediatric patients diagnosed with SARS-CoV-2 infection, the clinical symptoms of the disease were relatively mild, however the reoccurrence of SARS-CoV-2 was common even after two consecutive negative tests. While additional cohorts are needed to understand this phenomena, this study highlights the need to reconsider and improve existing prevention and control measures, particularly in children.

## Data Availability Statement

All datasets presented in this study are included in the article/supplementary material.

## Author Contributions

ZZ and LL conceptualized and designed the study, and reviewed and revised the manuscript. XL and JL designed the data collection instruments and drafted the initial manuscript. ZH, MH, and QC collected data, carried out the initial analyses, and reviewed and revised the manuscript. TX, LW, and HW completed blood sample collection and serum antibody detection. QH coordinated and supervised data collection, and critically reviewed the manuscript for important intellectual content. All authors approved the final manuscript as submitted and agreed to be accountable for all aspects of the work.

## Conflict of Interest

The authors declare that the research was conducted in the absence of any commercial or financial relationships that could be construed as a potential conflict of interest.

## Abbreviations

Ab: total antibody
Ab-ELISA: double-antigen sandwich enzyme-linked immunosorbent assay
COVID-19: coronavirus disease 2019
CMIA: Chemiluminescence Microparticle Immuno Assay
CT: computed tomography
CRP: Increasing serum c-reactive protein
IgM: sero-converted immunoglobulin-M
IgM-ELISA: IgM μ-chain capture method
IgG: immunoglobulin-G
IgA: immunoglobulin-A
IQR: interquartile range
IL-6: Increasing serum interleukin-6
RT-PCR: real-time reverse transcription polymerase chain reaction
RBD: receptor-binding domain
SARS-CoV-2: severe acute respiratory syndrome coronavirus 2
WHO: World Health Organization.
